# Impact of brain segmentation methods on regional metabolism quantification in ^18^F-FDG PET/MR analysis

**DOI:** 10.1186/s13550-023-01028-8

**Published:** 2023-09-05

**Authors:** Yi Shan, Shao-zhen Yan, Zhe Wang, Bi-xiao Cui, Hong-wei Yang, Jian-min Yuan, Ya-yan Yin, Feng Shi, Jie Lu

**Affiliations:** 1 Department of Radiology and Nuclear Medicine, Xuanwu Hospital, Capital Medical University, #45 Changchunjie, Xicheng District, Beijing, 100053 China; 2 Beijing Key Laboratory of Magnetic Resonance Imaging and Brain Informatics, Beijing, 100053 China; 3 Central Research Institute, United Imaging Healthcare Group, Shanghai, 201807 China; 4 Shanghai United Imaging Intelligence Co., Ltd., Shanghai, 200030 China

**Keywords:** Magnetic resonance imaging, Positron emission tomography, Metabolism, Artificial intelligence

## Abstract

**Background:**

Accurate analysis of quantitative PET data plays a crucial role in studying small, specific brain structures. The integration of PET and MRI through an integrated PET/MR system presents an opportunity to leverage the benefits of precisely aligned structural MRI and molecular PET images in both spatial and temporal dimensions. However, in many clinical workflows, PET studies are often performed without the aid of individually matched structural MRI scans, primarily for the sake of convenience in the data collection and brain segmentation possesses. Currently, two commonly employed segmentation strategies for brain PET analysis are distinguished: methods with or without MRI registration and methods employing either atlas-based or individual-based algorithms. Moreover, the development of artificial intelligence (AI)-assisted methods for predicting brain segmentation holds promise but requires further validation of their efficiency and accuracy for clinical applications. This study aims to compare and evaluate the correlations, consistencies, and differences among the above-mentioned brain segmentation strategies in quantification of brain metabolism in ^18^F-FDG PET/MR analysis.

**Results:**

Strong correlations were observed among all methods (*r* = 0.932 to 0.999, *P* < 0.001). The variances attributable to subject and brain region were higher than those caused by segmentation methods (*P* < 0.001). However, intraclass correlation coefficient (ICC)s between methods with or without MRI registration ranged from 0.924 to 0.975, while ICCs between methods with atlas- or individual-based algorithms ranged from 0.741 to 0.879. Brain regions exhibiting significant standardized uptake values (SUV) differences due to segmentation methods were the basal ganglia nuclei (maximum to 11.50 ± 4.67%), and various cerebral cortexes in temporal and occipital regions (maximum to 18.03 ± 5.52%). The AI-based method demonstrated high correlation (*r* = 0.998 and 0.999, *P* < 0.001) and ICC (0.998 and 0.997) with FreeSurfer, substantially reducing the time from 8.13 h to 57 s on per subject.

**Conclusions:**

Different segmentation methods may have impact on the calculation of brain metabolism in basal ganglia nuclei and specific cerebral cortexes. The AI-based approach offers improved efficiency and is recommended for its enhanced performance.

## Introduction

Accurate analysis of quantitative positron emission tomography (PET) data plays a crucial role in studying small, specific brain structures. For instance, investigations of abnormalities in the hippocampus in Alzheimer’s disease, localization of the epileptogenic zone in epilepsy, and delineation of lesion distribution in patients with brain tumors or ischemic stroke [1–4]. Magnetic resonance imaging (MRI) has high soft-tissue contrast and image resolution, facilitating localization and segmentation of brain regions of interest (ROIs) in individual subjects. Consequently, the integration of PET and MRI through an integrated PET/MR system presents an opportunity to leverage the benefits of precisely aligned structural MRI and molecular PET images in both spatial and temporal dimensions, thereby enhancing the value of such studies [5–8].

However, in many clinical workflows, PET studies are often performed without the aid of individually matched structural MRI scans, primarily for the sake of convenience in the data collection and brain segmentation possesses. Currently, two commonly employed segmentation strategies for brain PET analysis are distinguished: methods with or without MRI registration and methods employing either atlas-based or individual-based algorithms [9]. These strategies can be implemented using well-established brain automatic analysis tools, including statistical parametric mapping (SPM) [10], FMRIB software library (FSL) [11], and FreeSurfer [12]. While the previous studies have compared morphological parameters derived from structural MRI using these methods, none have specifically examined the impact of MRI segmentation when registered to PET, nor have they compared these results with regional standardized uptake values (SUV) obtained solely from PET data. Moreover, the development of artificial intelligence (AI)-assisted methods for predicting brain segmentations holds promise but requires further validation of their efficiency and accuracy for clinical applications [13, 14].

The present study aims to apply the three pairs of segmentation strategies to quantitative brain PET studies and assess their respective effects on calculating brain metabolism. To accomplish this, we employed the gold-standard tracer ^18^F-FDG, acquired precisely matched PET and MRI images using an integrated PET/MR system. The three pairs of segmentation methods considered are as follows: (1) methods with or without MRI registration employing SPM and denoted as “SPM_MRI_ATL” and “SPM_PET_ATL,” respectively; (2) atlas-based or individual-based methods with or without MRI registration using FSL, and referred to as “FSL _MRI_ATL,” “FSL _MRI _IND,” “FSL _PET_ATL,” and “FSL_PET_IND”; and (3) methods with or without AI assistance using FreeSurfer, and denoted as “Neural Network” and “FreeSurfer.” By comparing the relationships, consistencies, and differences between SUVs derived from these segmentation methods, we aim to elucidate whether these strategies significantly impact the calculation of brain metabolism. Furthermore, we hypothesize that specific brain regions may be influenced differently by various segmentation methods and postulate that AI-assisted approaches could offer time-saving benefits and thus be preferable for brain PET analysis.

## Materials and method

### Subjects and data acquisition

The brain PET/MR images of the 40 healthy volunteers (23 males and 17 females, age range: 31 to 66 years) were scanned using an integrated 3.0 T PET/MR scanner (uPMR 790; UIH). All subjects were injected intravenously with 3.7 MBq/kg ^18^F-FDG tracer. The MRI and PET list mode data were acquired 30 min (38 ± 11.5 min) after the injection, started with a 5-min scan of ultra-short echo time MRI sequence for PET attenuation correction. The structural MRI images were obtained using a three-dimensional (3D) T1-weighted (T1w) sequence (repetition time = 7.86 ms, echo time = 3.2 ms, inversion time = 750 ms, flip angle = 10°, number of slices = 288, voxel sizes = 1.0 × 1.0 × 1.0 mm^3^, matrix size = 256 × 230, echo train length = 160, scan time = 266 s). The PET images were reconstructed with the ordered subset-expectation–maximization (OSEM) algorithm during a 24-min scan (3 iterations and 20 subsets with time of flight (TOF) and point spread function (PSF), matrix size = 256 × 256, field of view (FOV) = 25.6 cm, voxel size = 1.0 × 1.0 × 1.0 mm^3^). SUVs were calculated by the default settings in the workstation as:

$$\mathrm{SUV}=\frac{\mathrm{radioactivity }(\mathrm{kBq}/\mathrm{ml})}{\mathrm{injected\, dose}\left(\mathrm{MBq}\right)*\mathrm{decay\, factor}/\mathrm{body\, weight }(\mathrm{kg})}$$

where decay factor = exp(− 0.693 × wait time/radionuclide half-life).

### Templates and atlases

Commonly used methods of brain segmentation are voxel-based morphometry (VBM) and surface-based morphometry (SBM), which have different templates and atlases. VBM adopts the ICBM152 template proposed by the International Consortium for Brain Mapping (ICBM) [15]. The standard MRI template, with a voxel size of 1 mm × 1 mm × 1 mm and a matrix of 182 × 182 × 218, is provided by FSL [11]. The standard PET template, with a voxel size of 1 mm × 1 mm × 1 mm and a matrix size of 182 × 200 × 215, was provided by Darkner Sune of the University of Copenhagen (https://staticcuris.ku.dk/portal/files/55751238/MNI152_PET_1mm.nii). SBM adopts the Desikan-Killiany atlas [16] proposed by Rahul S. Desikan et al., with a voxel size of 1 mm × 1 mm × 1 mm and a matrix size of 256 × 256 × 256. Thirty-two brain regions and 68 cortical brain regions were specified. Then, they were mapped to 21 whole-brain regions and 48 cortical brain regions of the Harvard–Oxford brain atlas.

### Brain segmentation process

**Fig. 1:**
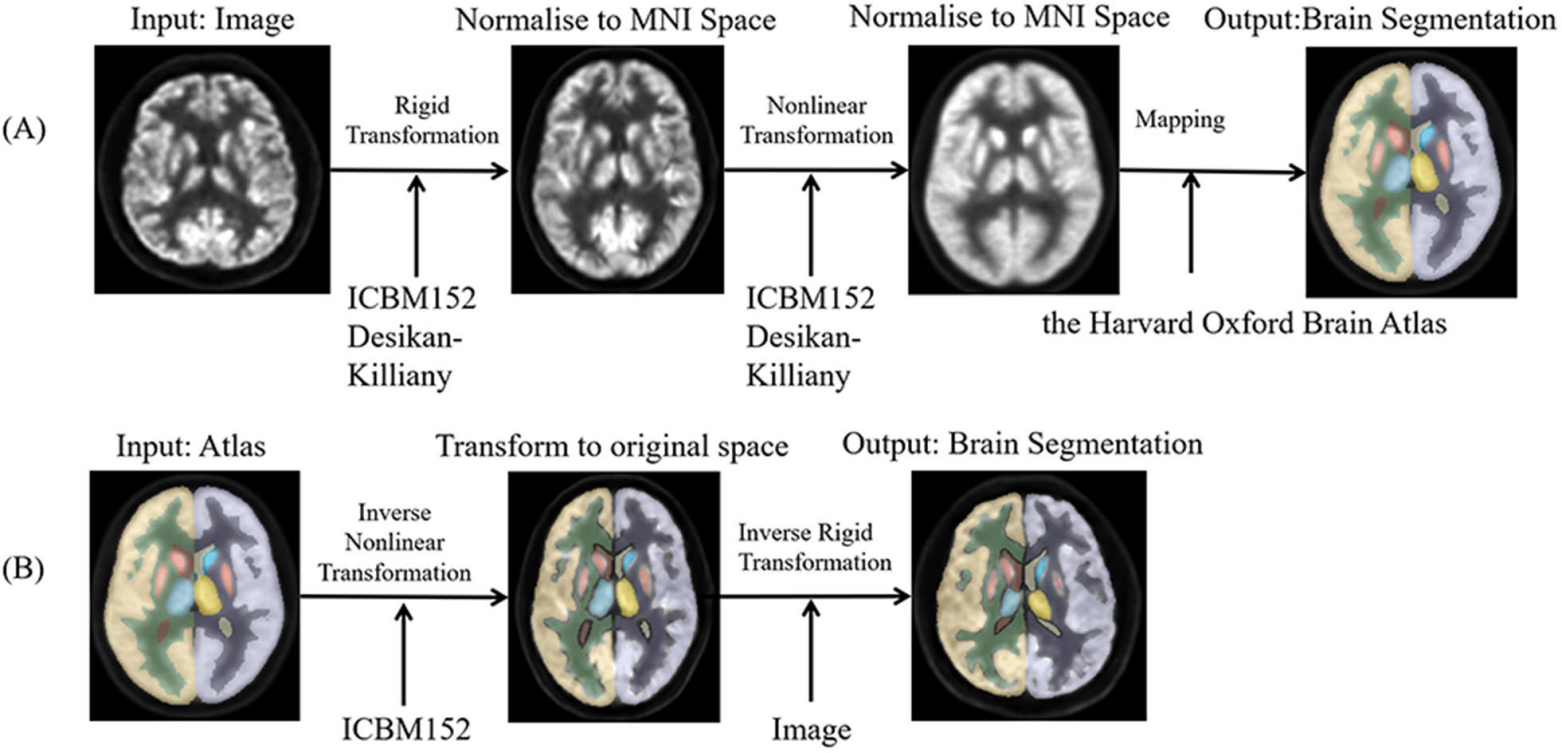
Block diagram of atlas-based (**A**) and individual-based (**B**) segmentation

In the atlas-based segmentation approach, the segmentation problem is reduced to an image registration problem. Image registration was performed in two steps (shown in Fig. 1A). First, the image to be segmented was rigidly registered to the standard brain template so that they are roughly aligned. Then, we used a nonlinear registration algorithm to complete the fine registration. We used large deformation differential homeomorphism [17] to register the new input image to the brain template, and the presegmented atlas was used to obtain the segmentation of the input image in the MNI152 space. Another method is individual-based segmentation (shown in Fig. 1B). The segmentation result obtained in the MNI152 space as described above is transformed to the original individual image space through the mathematical inverse transform operator to obtain the segmentation result of the original input image. It should be noted that this segmentation method is not done directly in the individual image space.

For the methods named “SPM_MRI_ATL” and “SPM_PET_ATL,” data processing was performed by using VBM-DARTEL [15] of SPM, based on MATLAB R2018b (MathWorks). First, we segmented the white matter (WM), gray matter (GM), and cerebrospinal fluid of each subject. The unified segmentation algorithm in SPM, which combined tissue classification, bias correction, and image registration in the same generative model, was used. Then, we created a brain template for this group of people (DARTEL template). Afterward, we normalized the images to Montreal Neurological Institute (MNI) space with smoothing under the parameter Gaussian full width at half maximum (FWHM) set to “8 8 8”. For the methods named “FSL_MRI_ATL,” “FSL_MRI_IND,” “FSL_PET_ATL,” and “FSL_PET_IND,” linear registration was completed by using FMRIB’s Linear Image Registration Tool of the FSL, and nonlinear registration was completed by using FMRIB’s Nonlinear Image Registration Tool of the FSL (http://fsl.fmrib.ox.ac.uk/fsl/fslwiki/). SIENAX from FSL was used to first extract brain and skull images from input data of a single full head. Images were then affine registered to the MNI152 space.

**Fig. 2:**
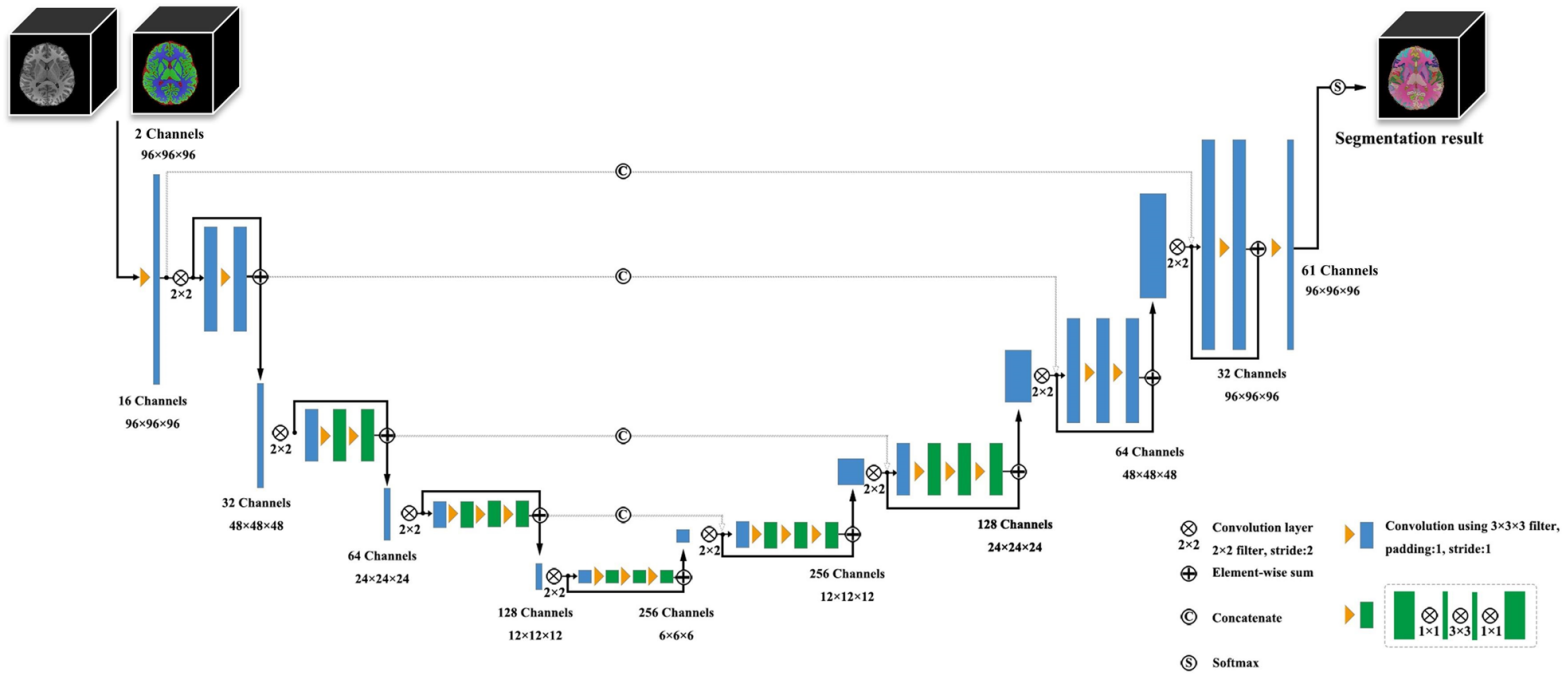
The architecture of the VB-Net method for brain ROI segmentation. VB-Net consists of two input channels, which are the original MRI images, and the segmentation maps of gray matter, white matter, and cerebrospinal fluid obtained from the VBM analysis of SPM. VB-Net consists of four levels with an encoding path followed by four levels of the corresponding decoding path. On the left side of the network, the encoding path reduces the size of the input by downsampling. The network was divided into four blocks, which comprise several convolutional blocks and residual blocks. A skip connection was introduced to improve the segmentations, and bottleneck layers were introduced to decrease the memory consumption. Similarly, on the right side of the network, the decoding path recovered the semantic segmentation image by deconvolution

The process of FreeSurfer includes intensity normalization, Talairach spatial registration, skull dissection, white matter segmentation, smoothing of mosaic surfaces, and automatic topology correction. The tessellated surface was used as the starting point of the deformable surface algorithm to find the white matter and then the pial boundary. For the method with assistance from AI named Neural Network, images were processed with a previously established VB-Net toolkit [18] in a standard pipeline, including intensity correction, AC-PC alignment, skull stripping, tissue segmentation, and brain parcellation [16]. This toolkit is a deep learning-based framework with encoder, decoder, and bottleneck layers, and the architecture of VB-Net is shown in Fig. 2. Over 1800 MRI images from the publicly available Consortium for Reliability and Reproducibility (CoRR) were used as training data [19]. See Table 1 for processor configurations.

**Table 1:** Server information

Server Information		
Hardware Environment	CPU	Intel(R) Xeon(R) CPU E5-2640 v4 @ 2.40 GHz
	Number of Cores	80 Cores
	RAM	252 GB
	Hard Disk	1 TB
Software Environment	Operating System	Linux Version 3.10.0-862.el7.x86_64
	Application	MATLAB 2018b

### Statistical analysis

All data were descripted as mean ± standard deviation. Pearson correlation analysis was used to determine the correlation coefficients between SUVs derived from different methods in all ROIs [20]. The homogeneity of variance was tested by Jarque–Bera test. Multiway analysis of variance was used to analyze the most possible variance source generating the variances in brain SUV calculations [21]. We considered three possible factors: SUVs from different volunteers, SUVs from different brain regions, and SUVs derived by using different brain segmentation methods. The intraclass correlation coefficient (ICC) was used to quantify the consistency among the three pairs of segmentation strategies. To measure the differences between each pair of the two given strategies (for example, methods with MRI registration as strategy A, while methods without MRI registration as strategy B), we calculated the percentage of difference value as follows: 2 × (SUV_A_-SUV_B_)/(SUV_A_ + SUV_B_) × 100%.

## Results

### Correlations between SUVs obtained by different segmentation methods

**Fig. 3:**
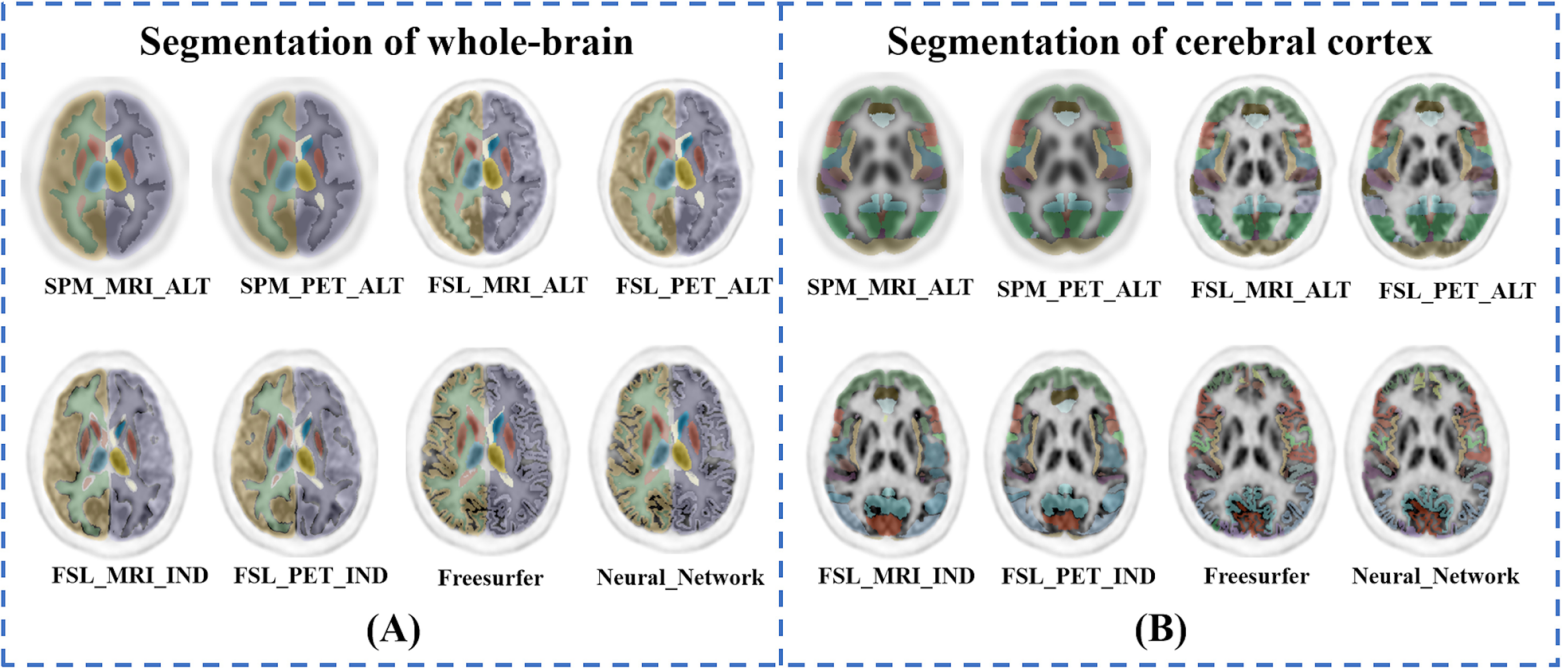
The segmentation results of different methods overlaid on the PET images of one subject. **A** The results at the whole-brain level, and **B** depicts the results at the cerebral cortex level. Different methods produced brain segmentations with similar appearances. The methods based on SPM and FSL divided the cortical brain regions into block-shaped areas that contain cerebrospinal fluid; in contrast, the method based on FreeSurfer and Neural Network divided the cortical brain regions into surface-shaped areas that do not contain cerebrospinal fluid

**Fig. 4:**
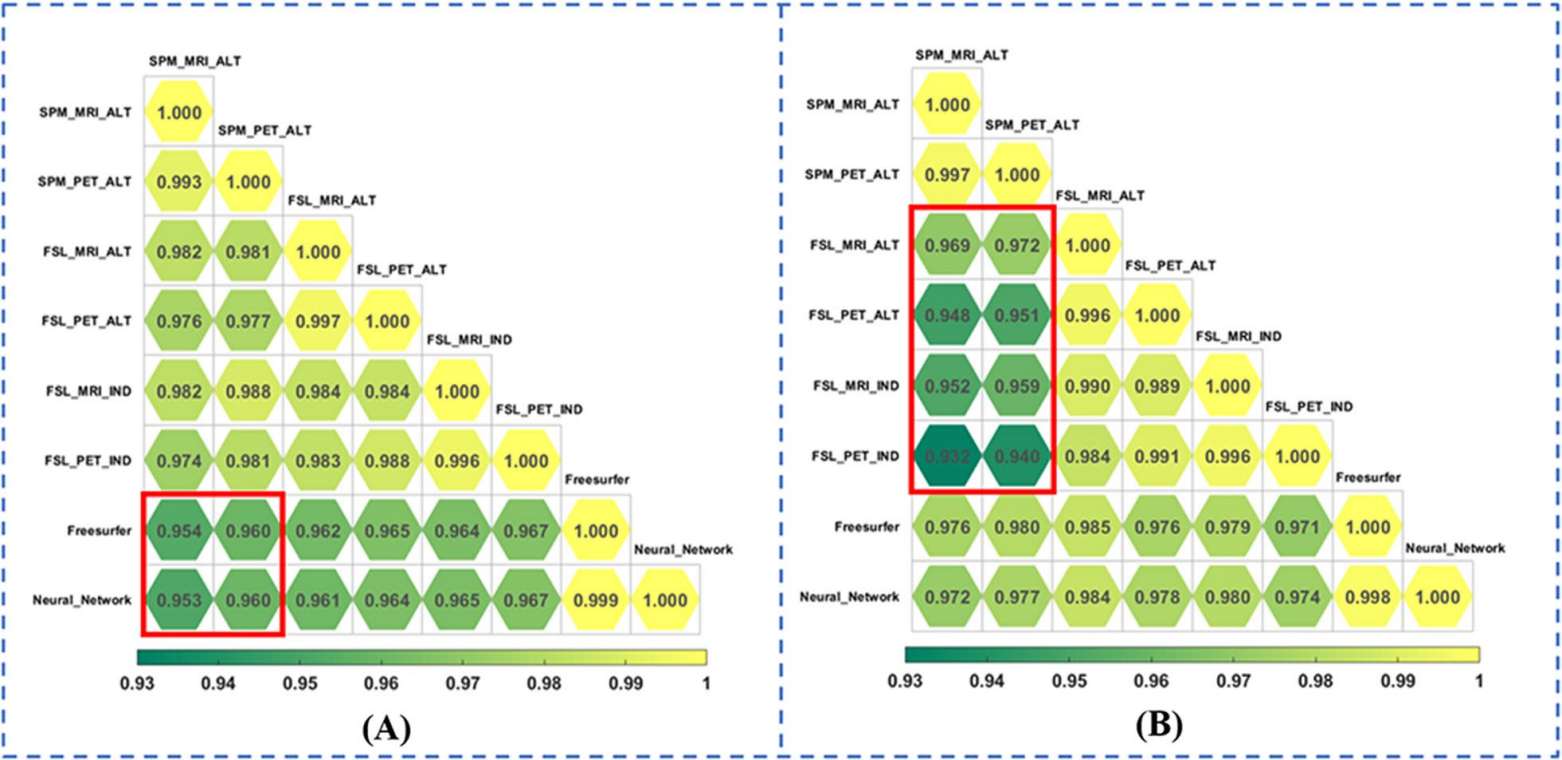
The Pearson correlation coefficients of SUVs calculated by eight different segmentation methods in the 21 brain regions at the whole-brain level (**A**) and 48 cortical regions at the cerebral cortex level (**B**)

Eight brain segmentation methods were performed and their performances were compared (shown in Fig. 3). Strong correlations among different methods of whole-brain segmentation (*r* = 0.953 to 0.999, *P* < 0.001) and cerebral cortex segmentation (*r* = 0.932 to 0.998, *P* < 0.001) are illustrated in Fig. 4. At the whole-brain level, comparatively weaker correlations (*r* = 0.953 to 0.960) were found among atlas-based SPM, individual-based FreeSurfer and Neural Network methods (highlighted in Fig. 4A), while at the cerebral cortex level, comparatively weaker correlations (*r* = 0.932 to 0.972) were found between SPM and FSL with either atlas- or individual-based methods (highlighted in Fig. 4B).

### Possible sources of variation in the calculation of brain SUVs

The possible sources of variation in the calculation of SUVs were explored (shown in Table 2). At the whole-brain level, the results showed that variances among brain regions (*F* = 26,140.6) > variances among subjects (*F* = 22,590.5) > variances among methods (*F* = 1003.1), while at the cortical brain level, the results showed that variances among subjects (*F* = 50,963.4) > variances among methods (*F* = 4233.2) ≈ variances among brain regions (*F* = 4098.6).

**Table 2:** Multifactor analysis of variance in the calculation of SUVs at the whole-brain and cerebral cortex levels

	Source	Sum Sq	*d.f*	Mean Sq	*F*	*P* value
Whole-brain level	Brain regions	29,234.7	20	1461.7	14,265.0	< 0.001
	Subjects	39,571.9	39	1014.7	9902.1	< 0.001
	Methods	857.3	7	122.5	1195.2	< 0.001
	Brain region × Subject	5103.8	780	6.54	63.9	< 0.001
	Brain region × Method	1947.4	140	13.9	135.7	< 0.001
	Subject × Method	163.6	273	0.6	5.8	< 0.001
	Error	559.5	5460	0.1		
	Total	77,438.1	6719			
Cerebral cortex level	Brain region	22,250.2	47	473.4	4261.8	< 0.001
	Subject	137,029.7	39	3513.6	31,630.8	< 0.001
	Method	3329.5	7	475.6	4282.0	< 0.001
	Brain region × Subject	6336.7	1833	3.5	31.1	< 0.001
	Brain region × Method	2164.6	329	6.6	59.2	< 0.001
	Subject × Method	682.8	273	2.5	22.5	< 0.001
	Error	1425.3	12,831	0.1		
	Total	173,218.8	15,359			

*Sum Sq.* sum of square; *d.f.* degree of freedom; *Mean Sq.* mean square; and *F F* value

### Consistencies and differences among the three pairs of segmentation strategies

For the above-mentioned three pairs of segmentation strategies, we evaluated the consistency among methods with or without MRI data registration, methods with atlas-based or individual-based algorithms, and methods with or without AI assistance using FreeSurfer. For methods with MRI (SPM_MRI_ATL, FSL_MRI_ATL, FSL_MRI_IND, FreeSurfer, and Neural Network) or without MRI (SPM_PET_ATL, FSL_PET_ATL, and FSL_PET_IND) registration, we further divided them according to whether their segmentation algorithm was atlas-based (SPM_MRI_ATL, FSL_MRI_ATL, SPM_PET_ATL, and FSL_PET_ATL) or individual-based (FSL_MRI_IND, FSL_PET_IND, FreeSurfer, and Neural Network), and vice versa.

As illustrated in Table 3, stronger ICCs between methods with MRI and without MRI registration were found at the whole-brain level (0.951 to 0.985) than those at the cerebral cortex level (0.924 to 0.975). The mean difference in SUVs among 21 whole-brain regions was 3.43 ± 2.31%. Brain regions with mean difference over 1 standard deviation from mean value (5.74%) were in the bilateral caudate (right, 8.54 ± 1.76%; left, 7.48 ± 1.57%) and right putamen (5.98 ± 1.16%). The mean difference in SUVs among 48 cerebral cortex regions was 3.01 ± 1.68%. Cortical regions with mean difference over 1 standard deviation from mean value (4.69%) were in the planum temporale (8.77 ± 2.50%), occipital fusiform gyrus (6.90 ± 2.18%), anterior division of the temporal fusiform cortex (6.64 ± 2.98%), planum polare (6.36 ± 2.67%), posterior division of the superior temporal gyrus (5.67 ± 1.66%), and frontal pole (5.20 ± 2.46%).

**Table 3:** The consistencies of regional brain SUVs among the three pairs of segmentation strategies at the whole-brain and cerebral cortex level

The consistencies among different methods		With or without MRI registration			With atlas- or individual-based algorithms			With or without AI
		With atlas-based algorithms	With individual-based algorithms	All	With MRI registration	Without MRI registration	All	
Whole-brain level	ICC	0.984	0.951	0.985	0.963	0.936	0.970	0.998
	LB	0.962	0.884	0.964	0.913	0.851	0.928	0.995
	UB	0.994	0.980	0.994	0.985	0.973	0.987	0.999
Cerebral cortex level	ICC	0.974	0.924	0.975	0.741	0.879	0.819	0.997
	LB	0.954	0.869	0.956	0.582	0.795	0.699	0.995
	UB	0.985	0.957	0.986	0.846	0.930	0.894	0.998

*ICC* intraclass correlation coefficients, *UB* and *LB* the upper bound and lower bound of 95% confidential interval

Higher ICCs between methods with atlas-based or individual-based algorithms were found at the whole-brain level (0.936 to 0.970) than at the cerebral cortex level (0.741 to 0.879). The mean difference in SUVs among 21 whole-brain regions was 6.04 ± 2.79%. Brain regions with mean difference over 1 standard deviation from mean value (8.83%) were in the bilateral caudate (right, 10.38 ± 5.07%; left, 11.50 ± 4.67%), cerebral cortex (right, 9.26 ± 1.94%; left, 10.15 ± 2.20%), and right lateral ventricle (10.90 ± 3.71%). The mean difference in SUVs among 48 cerebral cortex regions was 7.79 ± 3.85%. Cortical regions with mean difference over 1 standard deviation from mean value (11.64%) were in the anterior division of the temporal fusiform cortex (18.03 ± 5.52%), occipital pole (16.13 ± 4.40%), inferior and superior division of the lateral occipital cortex (14.33 ± 3.64%, 13.03 ± 3.88%), frontal pole (13.89 ± 6.68%), anterior division of the supramarginal gyrus (12.68 ± 2.85%), anterior division of the middle temporal gyrus (12.65 ± 2.71%), and pars triangularis of the inferior frontal gyrus (12.09 ± 3.00%).

The ICCs between methods with or without AI assistance using FreeSurfer were 0.998 and 0.997, respectively, at the whole-brain level and cerebral cortex level. The mean difference in SUVs among 21 whole-brain regions was 1.85 ± 1.09%; no region had a difference greater than 5%. The mean difference in SUVs among 48 cerebral cortex regions was 1.17 ± 0.53%; no region had a difference greater than 3%. We further compared the average time cost for analyzing one subject by using FreeSurfer and the “Neuronal Network.” The former required 8.13 h for one subject on average, while the latter required 57 s on average. The computation time cost for the training steps of “Neuronal Network” was about 3.5 days.

## Discussion

In our study, we conducted an analysis of brain metabolism using eight different segmentation methods with PET and MRI images acquired from integrated PET/MR system. While prior research has explored the impact of volumetric software (FSL vs. SPM vs. FreeSurfer) on MRI data, our study contributes by examining the potential influence of these morphological methods on PET analysis [22–25]. Overall, strong correlations were found among all methods at both the whole-brain and cerebral cortex levels, indicating their consistent performance and robust interrelationships. However, relatively weaker correlations were found between SUVs derived by VBM-based and SBM-based methods, as well as between SPM and FSL specially at the cerebral cortex level. Given that both SPM and FSL are VBM-based approaches, this finding suggests that VBM introduces greater variability in cortical PET calculations compared to SBM. Previous studies have indicated that while VBM can detect similar group differences as SBM in morphological studies, they may yield different regional statistics under certain conditions. SBM has demonstrated its suitability for capturing brain changes over time in longitudinal performance [26]. Additionally, the selection of analysis method (SPM vs. FSL vs. FreeSurfer) has been shown to impact different brain components [23]. For instance, in patients with multiple sclerosis, SBM exhibited greater robustness in GM volumes across different scanners compared to VBM and proved to be a viable alternative for WM segmentations [24]. In patients with cognitive impairment, all methods were capable of detecting decreased GM and WM volumes, while SBM yielded larger GM and smaller WM volumes than VBM [25]. Consequently, we propose that the choice of VBM-based or SBM-based methods may exert a limited yet specific influence to PET analysis.

Additionally, our findings highlight that variability attributed to subject and brain region factors surpassed that associated with the segmentation method. This finding underscores the necessity of employing individualized quantitative in brain metabolism research. Previous studies have emphasized the significance of considering individual differences among subjects and brain regions. For example, inter-subject variability of the cerebral cortex was significantly correlated with the degree of evolutionary cortical expansion and was also related to the variability in sulcal depth rather than cortical thickness [27]. The strength of association between brain function and structure also varied across the cortex [28]. Novel approaches have been developed to map brain activity at the individual level, effectively capturing the variability across subjects [29, 30]. Consequently, we suggest that it is especially crucial to select an SBM-based segmentation pipeline with MRI registration for PET studies, to minimize errors arising from individual differences.

Furthermore, our investigation revealed that brain regions demonstrating greater difference were located in the bilateral basal ganglia nuclei (especially in the caudate) and several cerebral cortexes in the temporal lobe, occipital lobe, and frontal lobe. Notably, differences resulting from utilization of individual atlas use (6.04 ± 2.79% to 7.79 ± 3.85%) exceeded those arising from MRI matching (3.01 ± 1.68% to 3.43 ± 2.31%). This finding bears considerable significance for clinical studies focusing on the physiological processes and pathological states within these above-mentioned regions. For example, the basal ganglia constitute essential components of complex distributed networks interconnected with specialized cortical areas, thereby contributing to distributed processing of higher-order cognitive functions and behaviors—an aspect of particular relevance in the context of neurodegeneration diseases [31]. The accumbens is involved in modulating information from the amygdaloid complex to the basal ganglia, mesolimbic dopaminergic regions, mediodorsal thalamus, and prefrontal cortex, which are all implicated in cognitive, emotional, and psychomotor functions [32]. Hence, we infer that it is necessary to calculate SUVs using methods incorporating precise structural registration and individual segmentation when examining the function of these brain regions.

In our analysis, FreeSurfer with or without AI assistance showed very high consistency at both the whole-brain level and cerebral cortex level, and the AI-based “Neuronal Network” shortened the processing time for analyzing one subject from an average of 8.13 h to 57 s. The clinical value of FreeSurfer has been verified in the detection of cortical abnormalities in patients with neurodegenerative diseases [13], schizophrenia [33, 34], bipolar disorder [35], and anisometropic amblyopia [36]. However, a notable drawback of FreeSurfer is extensive processing time, which is commonly range from 10–20 h for one subject, thereby greatly limiting its clinical applicability. Rapid AI-assisted postprocessing methods have been preliminarily employed in quantitative brain PET studies for the diagnosis of Parkinson’s disease and dementia subjects [37, 38]. Furthermore, deep learning techniques have been developed to predict FreeSurfer segmentations of MRI and have been established and applied in a model of Alzheimer’s disease [13, 14]. Our results further provide evidence for the high efficiency and clinical convenience of AI-based FreeSurfer methods in quantitative PET study. If this method could be applied to other populations in the future, it should continuously be optimized due to the increasing amount of training data.

As a limitation of our study, the small sample size and the lack of strict age restrictions for the subjects may have influenced the calculation of SUVs by different segmentation methods. The basic conditions of subjects (such as age and gender) may also influence SUV calculation of brain PET by different segmentation methods, which could be further verified by large-scale, multi-center, and longitudinal studies in the future. Additionally, inclusion of patients with abnormal metabolism in specific brain areas, such as Alzheimer’s disease or brain tumors, would help assess whether segmentation methods could influence the identification of lesions. Thus, the accuracy comparisons between different methods could be further discussed in specific population and clinical applications, such as subjects with various neurological conditions or age groups.

## Conclusions

In conclusion, our study evaluated various methods for analyzing brain metabolism using precisely matched PET and MRI data obtained from an integrated PET/MR system. We found relatively strong correlations between all methods. However, some method comparisons demonstrated insufficient consistency (with minimum correlation coefficient of 0.741) and significant differences (up to 18.03%) in several brain regions. Consequently, methods incorporating MRI registration (particularly those employing SBM) and individual-based algorithms may enhance the accuracy of SUV calculations in studies focusing on these regions. Furthermore, we presented a time-saving AI-based method that optimized SBM for PET/MR analysis. Our study has the potential to benefit radiologists and clinicians seeking more efficient and targeted methods for brain PET analysis.

## Data Availability

The datasets generated during the current study are available from the corresponding author on reasonable request.

## Abbreviations

SUV: Standardized uptake values
AI: Artificial intelligence
SPM: Statistical parametric mapping
FSL: FMRIB software library
ICC: Intraclass correlation coefficient
3D T1w: Three-dimensional T1-weighted
OSEM: Ordered subset-expectation–maximization
TOF: Time of flight
PSF: Point spread function
FOV: Field of view
VBM: Voxel-based morphometry
SBM: Surface-based morphometry
ICBM: International Consortium for Brain Mapping
GM: Gray matter
WM: White matter
MNI: Montreal Neurological Institute
FWHM: Full width at half maximum
